# Performance assessment of primary health care facilities in Brazil: Concordance between web-based questionnaire and in-person interviews with health personnel

**DOI:** 10.1371/journal.pone.0281085

**Published:** 2023-02-02

**Authors:** Luceime Olivia Nunes, Elen Rose Lodeiro Castanheira, Patricia Rodrigues Sanine, Marco Akerman, Maria Ines Baptistella Nemes

**Affiliations:** 1 Departament of Preventive Medicine, School of Medicine, University of São Paulo, São Paulo, São Paulo, Brazil; 2 Departament of Public Health, Medical School - Botucatu, São Paulo State University, Botucatu, São Paulo, Brazil; 3 Graduate Program in Public Health, Medical School - Botucatu, São Paulo State University, Botucatu, São Paulo, Brazil; 4 Department of Politics, Management and Health, School of Public Health, University of São Paulo, São Paulo, São Paulo, Brazil; Center for Primary Care and Public Health, SWITZERLAND

## Abstract

This study is a concordance analysis comparing answers to two external assessment tools for Primary Health Care (PHC) facilities that use two different data collection methodologies: (a) external assessment through structured interviews and direct observation of facilities conducted by the National Program for Improvement of Access and Quality of Primary Care (AE-PMAQ-AB), and (b) a computerized web-based self-administered questionnaire for Assessment of the Quality of Primary Health Care Services (QualiAB). The two surveys were answered by 1,898 facilities located in 437 municipalities in the state of São Paulo, Brazil, between 2017 and 2018. Both surveys aimed to assess the management and organization of PHC facilities. A total of 158 equivalent questions were identified. The answers were grouped by thematic similarity into nine domains: Territory characteristics; Local management and external support; Structure; Health promotion, disease prevention, and therapeutic procedures; Attention to unscheduled patients; Women’s health; Children’s health; Attention to chronic conditions; and Oral health. The results show a high level of concordance between the answers, with 81% of the 158 compared questions showing concordance higher than 0.700. We showed that the information obtained by the web-based survey QualiAB was comparable to that of the structured interview-based AE-PMAQ-AB, which is considered the gold standard. This is important because web-based surveys are more practical and convenient, and do not require trained interviewers. Online assessment surveys can allow immediate access to answers, reports and guidelines for each evaluated facility, as provided by the QualiAB system. In this way, the answers to this type of survey can be directly employed by users, allowing the assessment to fulfill all phases of an assessment process.

## Introduction

High-quality primary health care (PHC) can improve population and individual outcomes while reducing total health care costs [1], but quality in PHC has not been fully achieved in most countries [2–4]. Therefore, PHC quality assessment takes on special importance for prompting actions that improve health care team training, favoring change processes in the organization of care practices [5, 6].

Health facility assessment can be based on different methodologies and data collection approaches, all of which have advantages and drawbacks. To choose the best strategy, it is necessary to consider the purpose of the assessment, the themes that guide it, and its theoretical framework, as well as the analysis of its feasibility, reliability, utility, and access to its results by the facilities that took part in the assessment [7].

Assessments carried out through online surveys can be questioned in terms of confidentiality, confidentiality maintenance, response rate and sample representativeness [7–9]. On the other hand, they do offer several advantages. In addition to suitably fitting with pandemic contexts, such as the COVID-19 one, they show high feasibility as they allow large-scale surveys, can be conducted in a short period of time, offer a good cost-benefit ratio, and allow quick access to results by assessment researchers and users, thus constituting an important assessment survey tool [8, 9].

Data collection through in-person interviews in health facility assessment is a qualitative strategy that can be used in surveys. This type of survey is costlier and tends to use structured instruments, such as questionnaires or checklists. This more structured form of collection differs from approaches based on interviews that seek to expand explanatory power through the analysis of the facilities’ daily experience and the relationships established between teams and between teams and users [10].

In Brazil, PHC health facility assessment processes have been carried out using different instruments, with different methodologies, geographic coverage, and assessment focus [11–14]. In general, these are cross-sectional studies aimed at professionals and/or users based on in-person interviews using structured questionnaires in paper or in digital format, installed on tablets or smartphones that upload the data, or even in the form of online self-response. No studies have been conducted to compare information gathered through the different methods used, often similar in aim and data collection methods. The advance of information technology and the high cost of in-person interview surveys, especially in a continent-sized country like Brazil, has motivated the investigation into the correspondence between responses collected via web-based self-administered questionnaires and those collected through in-person structured interviews.

The institutionalization of assessment processes in Brazilian PHC facilities was consolidated with the implementation of the National Program for Improvement of Access and Quality of Primary Care—PMAQ-AB, which completed its third assessment cycle in 2018. Based on voluntary participation, the program awarded financial incentives that varied according to facility performance. Three assessment cycles were carried out throughout the country, by means of integrated steps that used data from health information systems, self-assessment instruments, and external assessments made *in loco* by trained interviewers (AE-PMAQ-AB). The criteria and standards used were based on the guidelines and protocols of the National Primary Care Policy (PNAB) and on the ethical and political principles of the Brazilian Unified Health System (SUS) [15, 16].

In this context of encouragement of PHC facility assessment, the system for Assessment of Primary Health Care Services (QualiAB) was created in the state of São Paulo, Brazil. It is a computerized system using a web-based self-administered questionnaire, with assessment criteria and standards following the PNAB and SUS guidelines; adherence is voluntary and not linked to financial incentives. Eight QualiAB surveys have been conducted in different regions of the country. In the state of São Paulo, the last survey was conducted in 2017, preceding the AE-PMAQ-AB in the state by a few months [17].

By focusing on a particular fraction of the complex aspects involved in health facility assessment, this study investigates whether there is concordance between the responses to two assessment surveys that used structured questionnaires and adopted different data collection methodologies: the PMAQ-AB external assessment (AE-PMAQ-AB), conducted by in-person interviewers and considered here as the “gold standard,” and the QualiAB external assessment, conducted through a computerized web-based self-response system.

## Materials and methods

This is a concordance analysis study comparing answers of 1,855 PHC facilities to two surveys conducted with the aid of structured instruments in the state of São Paulo, Brazil, in 2017–2018. The two surveys consist in external assessments that use different information collection methods: the AE-PMAQ-AB assessment is conducted by in-person interviewers, whereas the QualiAB assessment uses a web-based self-response questionnaire.

The selection of assessment surveys was based on the comparability between answers, according to the equivalence of the following criteria: 1) focus on the organization of the work process; 2) use of the same technical and political references to define the criteria and standards of their indicators; 3) use of structure and process indicators; 4) occurrence in periods close to each other; and 5) assessment of PHC facilities in the same region.

The assessments were carried out at PHC facilities in the state of São Paulo, which has a municipally managed network of public PHC facilities covering approximately 60% of the population during the period studied. The state has 45.5 million inhabitants (21.9% of the Brazilian population) and has the country’s second highest Human Development Index (HDI): 0.783. However, its 645 municipalities display great geographical, populational, and socioeconomic heterogeneity. Forty percent of the municipalities have fewer than 10,000 inhabitants, are geographically distributed across coastal and mountainous regions that are poorly accessible, and have municipal HDIs between 0.862 and 0.639, thus presenting inequalities similar to Brazil’s [18–20].

The AE-PMAQ-AB assessment under analysis was conducted in the state of São Paulo between May and August 2018, as part of the third PMAQ-AB cycle. It was the result of a partnership between the Ministry of Health and public higher education institutions responsible for selecting, training, and hiring the university-level professionals who conducted the AE-PMAQ-AB data collection *in* loco16. The interviewers formed regionalized teams supervised by a coordinator responsible for planning their travel itinerary and checking the recorded information. Data collection was carried out through structured questionnaires installed on tablets equipped with a computerized system that sends the data to the Ministry of Health at the end of each interview. In the state of São Paulo, 2,693 family health teams based in 564 municipalities were assessed *in loco*. The municipal participation rate was 87.4%. No data are available on the total number of PHC facilities in the municipalities participating in the PMAQ assessment. Participation was encouraged by the Ministry of Health and partner institutions through presentations of the project in regional meetings and financial incentives to participate [21]. The Ministry of Health was slow in disclosing the end results of the PMAQ assessment. The final score determined the ranking of each facility and the amount of financial incentive for performance. The score was composed of the results achieved in three stages: (1) implementation of self-assessment procedures, accounting for 10% of the score; (2) assessment of contractual indicators, corresponding to 30%; and (3) the AE-PMAQ-AB assessment, which was the last stage and accounted for 60% of the final score [21]. The partial results relative to the AE-PMAQ-AB stage were not disclosed to the participants.

The QualiAB survey was conducted in the state of São Paulo between May and November 2017 as part of a research project resulting from the partnership of two public higher education institutions (UNESP and USP) and the São Paulo State Department of Health (SES SP). It was supported by the Council of Municipal Health Secretaries of the state of São Paulo (COSEMS SP) and encompassed 2,739 PHC facilities located in 514 municipalities in the state of São Paulo. A total of 79.7% of municipalities participated in the QualiAB survey, with a participation rate of 88.2% for the PHC facilities located in the participating municipalities. The QualiAB assessment project was presented by the SES SP to municipal health secretaries in regional meetings to encourage municipal participation. The other stages of the assessment were carried out via web: enrollment of the municipalities with password definition; enrollment of facilities by local managers with individualized access passwords; questionnaire response; and hierarchized access to results and standards according to the institutional rank of the participants. The computerized system provided participants with immediate access to their score and performance level, measured both globally and by indicator, as well as access to recommendations according to the criteria and standards used. There was no financial incentive to participate in the QualiAB assessment [22].

Both questionnaires were written in Portuguese, Brazil’s official language, and answered by two different professionals of each PHC facility. The content of the questionnaires used in both assessments was analyzed at two different times by two researchers, who identified 158 comparable questions. Even though these questions are not identical, they have equivalent phrasings, as they address the same aspects of work organization. The QualiAB answer alternatives tend to be more detailed, while in the AE-PMAQ-AB assessment, they are overall dichotomous (yes/no), as shown in Table 1. To allow comparison, the equivalence was established through the presence, or lack thereof, of paired items.

**Table 1:** An example of paired questions from the AE-PMAQ-AE and QualiAB data collection instruments. Brazil, 2017/2018.

AE-PMAQ-AB structured interviews	QualiAB web-based survey
Is the team’s coverage area defined?	The facility’s coverage area is defined:
	***Choose only one alternative***
□ Yes	□ 1) Administratively according to the central level of the Health Secretariat or other municipal health agency
□ No	□ 2) Through participative planning, considering the local reality and ease of access
	□ 3) In practice, the team defines an area to carry out actions in the community
	□ 4) Undefined coverage area
To compare the item “Defined coverage area”, AE-PMAQ-AB questions and QualiAB questions were used, which inquired about the existence or not of a defined coverage area, regardless of how it has been defined.	
Vaccines at the health facility	The following vaccines are administered at the facility:
General—Always available hepatitis B	
□ Yes	
□ No	
General—Always available HPV	
□ Yes	***Choose one or more alternatives***
□ No	□ Hepatitis B
General—Always available pentavalent	□ Pentavalent (DTP + Hib + HB)
□ Yes	□ Human Rotavirus
□ No	□ HPV (Human Papilloma Vírus)
General—Always available oral human rotavirus vaccine	
□ Yes	
□ No	
Service offerings	The care provided to people living with HIV comprise the following actions:
	***Choose one or more alternatives***
	□ Delivery of care to clinically stable and immunologically preserved HIV patients
	□ Existence of a specialized team for treating HIV/AIDS patients
Does the team provide care to people living with HIV/AIDS?	□ Active search for absent patients when requested by the local HIV/AIDS care facility
□ Yes	□ Delivery of care for acute complaints and chronic conditions, vaccination, prenatal testing, and preventive cancer protocols
□ No	
	□ The facility does not provide care to HIV patients, who receive care at specialized HIV/AIDS health facilities

The 158 comparable questions were grouped into nine domains relative to territory, structure, management, and attention to main demand and programs, as provided in the Brazilian National Primary Care Policy [23]. The domains comprehend different aspects of the organization of PHC facilities and make it possible to analyze concordance differences between facilities. The nine domains are: 1. Territory characteristics; 2. Local management and external support; 3. Structure; 4. Health promotion, disease prevention, and therapeutic procedures; 5. Attention to unscheduled patients; 6. Women’s health; 7. Children’s health; 8. Attention to chronic conditions; 9. Oral health.

After the comparable questions were identified, the facilities that responded to the two external assessments in the state of São Paulo were paired, resulting in a total of 1,855 facilities located in 434 municipalities.

The AE-PMAQ-AB assessment was used as the gold standard to validate the answers. This choice is justified because the AE-PMAQ-AB assessment was part of PMAQ, an official program of the Brazilian government for assessing PHC facilities during the period analyzed in this study, and because its criteria, standards, and indicators reflected the technical and political proposals for PHC facilities [16, 23]. That choice is also corroborated by the fact that PMAQ has nationwide reach and defines a "baseline" for PHC assessment in Brazil, which is confirmed by the multiple studies and assessments based on AE-PMAQ-AB data [14, 24–27].

The proportion of similar answers to the comparable questions from the two questionnaires was calculated, which made it possible to calculate sensitivity (TP/(TP+FN)), specificity (TN/(FP+TN)), and accuracy ((TP+TN)/N). TP stands for "true positive" (where both questionnaires have an affirmative answer for the same comparable question); FN stands for "false negative" (affirmative answer in QualiAB, negative in AE-PMAQ-AB); TN is "true negative" (negative answers in both questionnaires); FP is "false positive" (negative answer in QualiAB, affirmative in AE-PMAQ-AB); and N is the total of answers. The concordance between answers was analyzed with the Kappa coefficient test; all analyses were performed using the SPSS 21^®^ statistical package.

This study complied with the ethical principles laid out in Brazilian legislation, according to Resolution No. 446/2012 on the guidelines and regulations for medical research on human beings, and garnered approval from the ethics committee (Resolution No. 2.532.658, CAAE: 83479417.7.0000.5411, March 8, 2018).

## Results

**Fig 1:**
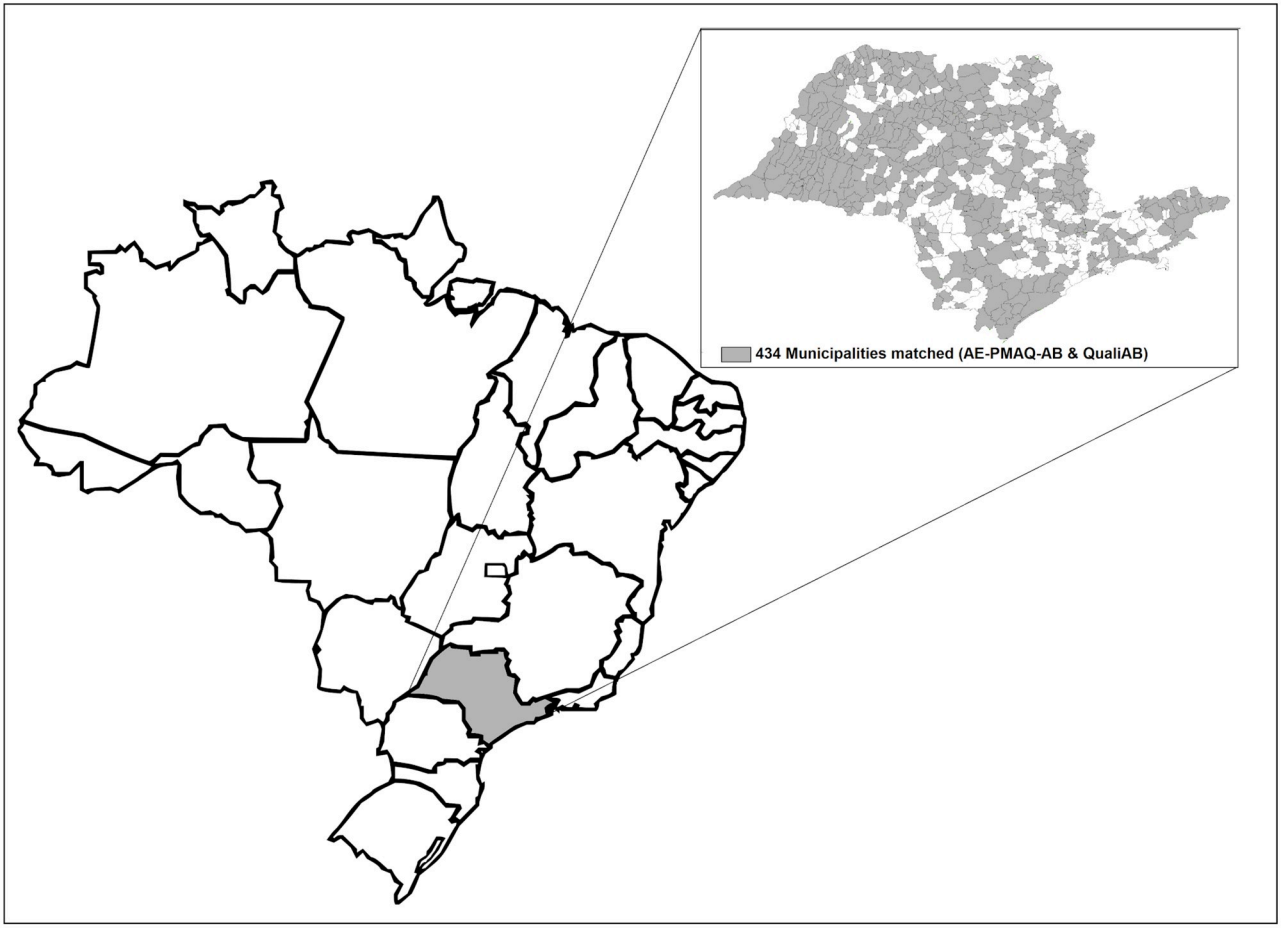
Geographic distribution of the 434 municipalities participating in the QualiAB, 2017 and AE-PMAQ-AB, 2018 assessments. Source: authors; Datasus software.

The pairing of questions from the two external assessments made possible the analysis of the concordance of the answers from 1,855 PHC facilities, geographically distributed across 67% of the municipalities of the state of São Paulo (Fig 1).

The grouping of questions into nine domains allowed for the analysis of the main aspects involved in the organization of the PHC facilities. A high concordance level was found between the answers to both assessments: 81% (128) of the QualiAB answers showed a concordance level higher than 0.700 in relation to the AE-PMAQ-AB answers. Only in the question addressing treatment of people living with HIV/AIDS was the concordance level lower than 0.500 (Acc = 0,414) (Table 2).

**Table 2:** Comparison of the answers to the QualiAB and the AE-PMAQ-AB questionnaires relative to territory characteristics, structure, and management and external support, according to accuracy, sensitivity, specificity, confidence interval, and Kappa coefficient. Brazil, 2017/2018.

**Domain: Territory Characteristics**							
**Subdomain: Territory Characteristics**	**Sens**	**Spec**	**Acc**	**CI95**		**Kappa**	**p**
Coverage area is defined	0.991	0.067	**0.981**	0.971	0.987	0.058	0.035
**Populations in the coverage area:**							
*Quilombolas*	0.692	0.996	**0.993**	0.989	0.998	0.679	<0.001
Indigenous populations	0.438	0.987	**0.980**	0.972	0.988	0.403	<0.001
Riverside populations	0.286	0.980	**0.968**	0.959	0.978	0.188	<0.001
Gypsies and urban nomads	0.387	0.962	**0.950**	0.935	0.960	0.223	<0.001
Campers and settlers	0.486	0.930	**0.920**	0.898	0.929	0.191	<0.001
**Domain: Structure**							
**Human Resources**	**Sens**	**Spec**	**Acc**	**CI95**		**Kappa**	**p**
Community health agent	0.997	0.729	**0.987**	0.981	0.993	0.780	<0.001
Occupational therapist	0.167	0.984	**0.969**	0.961	0.977	0.126	<0.001
**Physical Structure**							
Dedicated vaccination room	0.949	0.540	**0.833**	0.815	0.851	0.475	<0.001
Restroom adapted for persons with disabilities (PWD)	0.868	0.685	**0.810**	0.791	0.828	0.501	<0.001
Clinical consulting rooms with attached toilet	0.891	0.677	**0.787**	0.662	0.707	0.558	<0.001
Proper ventilation and lighting	0.728	0.595	**0.723**	0.708	0.751	0.082	<0.001
Dedicated wound-dressing room	0.882	0.443	**0.694**	0.612	0.658	0.342	<0.001
Dedicated room for sterilization/storage of sterilized material	0.966	0.216	**0.681**	0.767	0.806	0.160	<0.001
Electronic medical records	0.523	0.816	**0.653**	0.618	0.670	0.325	<0.001
**Material Resources**							
Gynecological examination table	0.985	0.500	**0.982**	0.976	0.989	0.157	<0.001
Internet access	0.955	0.793	**0.945**	0.937	0.958	0.615	<0.001
Wheelchair	0.960	0.348	**0.909**	0.895	0.923	0.280	<0.001
Dedicated vaccine refrigerator	0.911	0.256	**0.553**	0.518	0.566	0.156	<0.001
Male condom	0.994	0.028	**0.937**	0.925	0.949	0.037	0.008
**Domain: Local Management and External Support**							
Presence of a facility manager	0.973	0.133	**0.963**	0.953	0.971	0.079	0.001
Availability of user communication/complaint channels	0.993	0.081	**0.941**	0.928	0.953	0.118	<0.001
Holding of team meetings	0.990	0.038	**0.973**	0.961	0.979	0.035	0.166
External multiprofessional team support for addressing complex cases	0.926	0.143	**0.922**	0.905	0.935	0.009	0.507

Key: Sens = sensitivity; Spec = specificity; Acc = accuracy; CI = confidence interval; p = p-value. Source: QualiAB (2017); AE-PMAQ-AB (2018).

Table 2 comprehends domains relative to territory, some of the available resources, and management characteristics, showing high accuracy for nearly all compared questions, and varying sensitivity and specificity.

In Table 3, all the questions show accuracy higher than 0.900. While sensitivity is high for all three subdomains (health promotion, disease prevention, and therapeutic procedures), specificity is considerably lower for the subdomain Therapeutic Procedures.

**Table 3:** Comparison of the answers to the QualiAB and the AE-PMAQ-AB questionnaires relative to health promotion, disease prevention, and therapeutic procedures, according to accuracy, sensitivity, specificity, confidence interval, and Kappa coefficient. Brazil, 2017/2018.

Domain: Health Promotion, Disease Prevention, and Therapeutic Procedures							
	Sens	Spec	Acc	CI95		Kappa	p
**Health Promotion and Primary Prevention**							
Educational strategies for health promotion	0.990	0.088	**0.969**	0.954	0.974	0.101	0.523
**Vaccination**							
Human rotavirus	0.951	0.864	**0.931**	0.919	0.943	0.805	<0.001
HPV	0.950	0.855	**0.929**	0.916	0.941	0.788	<0.001
Hepatitis B	0.952	0.843	**0.927**	0.915	0.940	0.795	<0.001
Pentavalent	0.954	0.827	**0.923**	0.912	0.938	0.790	<0.001
Inactivated polio vaccine 1, 2 and 3 (IPV)	0.944	0.851	**0.922**	0.910	0.935	0.787	<0.001
DTaP	0.940	0.854	**0.920**	0.907	0.933	0.772	<0.001
Triple viral	0.938	0.826	**0.913**	0.899	0.927	0.743	<0.001
Double adult—diphtheria and tetanus (DT)	0.949	0.792	**0.911**	0.900	0.927	0.754	<0.001
Hepatitis A	0.920	0.851	**0.903**	0.892	0.920	0.744	<0.001
Pneumococcal 10	0.923	0.831	**0.901**	0.889	0.918	0.736	<0.001
**Therapeutic Procedures**							
Fingerstick glucose test (glucometer)	0.979	0.500	**0.974**	0.931	0.992	0.262	<0.001
Stitch removal	0.983	0.308	**0.976**	0.968	0.984	0.155	<0.001
Intramuscular injection	0.967	0.300	**0.961**	0.951	0.972	0.067	0.001

Key: Sens = sensitivity; Spec = specificity; Acc = accuracy; CI = confidence interval; p = p-value.

Source: QualiAB (2017); AE-PMAQ-AB (2018).

Table 4 shows low accuracy for the question about risk assessment of unscheduled patients, as well as for the questions in the subdomain Attention to Chronic Communicable Conditions, except for the question about bacilloscopy for tuberculosis. In the subdomain of Chronic Non-communicable Conditions, the questions about ocular fundus examination and mental health care also show low accuracy. The item with the lowest accuracy, "Delivery of care to persons living with HIV/AIDS", contrasts with the high accuracy seen in consultations for persons with diabetes and hypertension, as well as in the delivery of care to women and children.

**Table 4:** Comparison of the answers to the QualiAB and the AE-PMAQ-AB questionnaires relative to the delivery of care to unscheduled and scheduled patients (women, children, chronic conditions, oral health) according to accuracy, sensitivity, specificity, confidence interval, and Kappa coefficient. Brazil, 2017/2018.

**DOMAIN: Delivery of Care to Unscheduled Patients**							
**Unscheduled patients**	**Sens**	**Spec**	**Acc**	**CI95**		**Kappa**	**p**
Delivery of care to unscheduled patients	0.998	0.000	**0.996**	0.993	1.000	0.120	<0.001
Risk assessment of unscheduled patients	0.558	0.551	**0.555**	0.509	0.563	0.190	0.017
**DOMAIN: Women’s Health**							
	**Sens**	**Spec**	**Acc**	**CI95**		**Kappa**	**p**
**Gynecological cancer prevention**							
Mammogram	0.997	0.000	**0.995**	0.991	0.999	0.236	<0.001
Cytopahological sample collection	0.992	0.600	**0.991**	0.986	0.996	0.396	<0.001
**Prenatal care**							
Prenatal care delivery in the facility	0.986	0.437	**0.956**	0.945	0.967	0.466	<0.001
Fasting blood glucose test	0.994	0.078	**0.963**	0.951	0.972	0.115	<0.001
ABO-Rh test	0.994	0.057	**0.960**	0.947	0.969	0.080	<0.001
Toxoplasmosis serology	0.997	0.018	**0.960**	0.948	0.970	0.026	0.091
Obstetric ultrasonography	0.986	0.114	**0.960**	0.947	0.969	0.126	<0.001
First-morning urine test	0.998	0.000	**0.960**	0.949	0.971	0.066	0.008
Hemoglobin and hematocrit levels	0.997	0.000	**0.955**	0.944	0.967	0.075	<0.001
Hepatitis B serology	0.982	0.080	**0.952**	0.935	0.959	0.077	0.002
Syphilis serology (VDRL)	0.966	0.061	**0.936**	0.921	0.948	0.026	0.307
HIV serology	0.969	0.065	**0.932**	0.917	0.944	0.038	0.141
Oral glucose tolerance test	0.590	0.378	**0.576**	0.548	0.602	-0.009	0.529
Tests are not requested for pregnant women	0.086	0.999	**0.977**	0.967	0.984	0.145	<0.001
Pregnant woman’s vaccination is advised/monitored	0.973	0.431	**0.931**	0.913	0.942	0.456	<0.001
Pregnant woman’s healthcare card is required	0.951	0.500	**0.922**	0.906	0.935	0.406	<0.001
High-risk pregnancy screening	0.980	0.333	**0.912**	0.865	0.900	0.400	<0.001
**DOMAIN: Children’s Health**							
	**Sens**	**Spec**	**Acc**	**CI95**		**Kappa**	**p**
Childcare consultation for children aged up to 2 years (growth/development)	0.979	0.214	**0.943**	0.929	0.954	0.233	<0.001
Incentive to exclusive breastfeeding	0.955	0.259	**0.930**	0.907	0.936	0.175	<0.001
Incentive to continuous breastfeeding and introduction of healthy food at six months of age	0.953	0.263	**0.935**	0.914	0.942	0.141	<0.001
**DOMAIN: Attention to Chronic Conditions**							
**Attention to Chronic Non-communicable Conditions**							
Medical consultation for persons with hypertension	0.999	1.000	**0.999**	0.998	1.000	0.449	<0.001
Medical consultation for persons with diabetes	1.000	0.000	**0.998**	0.996	1.000	0.666	<0.001
Periodic glycated hemoglobin (HbA1c or A1c) monitoring	0.951	0.032	**0.932**	0.913	0.941	-0.010	0.663
Periodic ocular fundus examination for persons with type 2 diabetes	0.574	0.605	**0.599**	0.572	0.625	0.126	<0.001
Assistance by mental health professionals	0.481	0.787	**0.541**	0.511	0.565	0.160	<0.001
**Attention to Chronic Communicable Conditions**							
Delivery of care to persons living with HIV/AIDS	0.374	0.647	**0.414**	0.385	0.439	0.009	0.576
Bacilloscopy for tuberculosis	0.953	0.000	**0.944**	0.929	0.955	-0.015	0.417
The number of persons with leprosy is recorded	0.676	0.445	**0.596**	0.567	0.621	0.119	<0.001
Diagnosis of new leprosy cases	0.819	0.383	**0.594**	0.565	0.619	0.199	<0.001
Active contact tracing for leprosy	0.864	0.302	**0.593**	0.566	0.619	0.162	<0.001
Active search for leprosy treatment absentees	0.881	0.296	**0.588**	0.560	0.614	0.177	<0.001
Compulsory notification of leprosy cases	0.870	0.300	**0.587**	0.556	0.609	0.170	<0.001
**DOMAIN: Oral Health**							
	**Sens**	**Spec**	**Acc**	**CI95**		**Kappa**	**p**
Patient information is registered on medical record	0.944	0.173	**0.584**	1.000	1.000	0.080	<0.001
Deciduous tooth extraction (exodontia)	0.950	0.000	**0.940**	0.924	0.956	0.062	0.053
Resin dental fillings	0.918	0.111	**0.909**	0.890	0.929	0.017	0.433
Ulotomy/Ulectomy	0.467	0.744	**0.516**	0.480	0.548	0.111	<0.001
Oral cancer prevention and diagnosis strategies	0.924	0.250	**0.914**	0.895	0.933	0.042	0.065

Key: Sens = sensitivity; Spec = specificity; Acc = accuracy; CI = confidence interval; p = p-value.

Source: QualiAB (2017); AE-PMAQ-AB (2018).

The high accuracy combined with low, even negative, Kappa values is explained by the vulnerability of the Kappa coefficient test against marginal distributions and asymmetric joint distributions, since too high concordances, without a normal distribution, compromise Kappa values [27].

## Discussion

The results presented a high concordance level between answers to the paired questions of both assessments, showing that web-based questionnaires are a viable tool to assess work organization in PHC facilities when it comes to structure and processes.

The highest accuracy level was found in the more traditional actions of the Brazilian PHC programs, especially in relation to questions of health promotion, disease prevention, therapeutic procedures, definition of coverage area, local management, and health care programs such as care delivery to women, children, persons with hypertension and type 2 diabetes, and more traditional oral health procedures.

The lowest-accuracy questions may point to a lack of clarity in their formulation, respondents’ limited knowledge of the subject, or recent implementation of the service in question. For example, the item “Dedicated vaccine refrigerator” showed low accuracy level (0.533), which may be linked to the lack of clarity in the question’s phrasing. Neither the QualiAB nor the AE-PMAQ-AB questionnaire specifies the type of refrigerator (for home or commercial use), which may have led to diverging interpretations, as it is recommended to replace home-use refrigerators with ones that meet safety and quality standards [28].

The lowest accuracy level among the 158 paired questions was found in “Delivery of care to persons living with HIV/AIDS” (0.414), which has only recently been incorporated into the Brazilian PHC [29], followed by “Ulotomy/Ulectomy” (0.516), which are low-demand procedures unknown to many team members [30]. It is important to point out that the AE-PMAQ-AB questionnaire should be answered either by the facility manager or by the head of each department, or even by a doctor, whereas the QualiAB questionnaire should preferably be answered in a team meeting. It behooved the facility personnel to find the best way to answer the questionnaires without compromising service to patients. They were, therefore, answered by different team members of each facility, which may have influenced the answers regarding low-demand or recently implemented services.

Other not fully implemented services, such as electronic medical records, risk assessment protocols for persons with diabetes and hypertension, and mental health care, also showed low accuracy levels, which may be related to the reorganization of the facilities during that period.

In general, the questions showed lower specificity, i.e., higher discordance in relation to negative answers. In addition to the possible reasons mentioned above, this discrepancy may be due to the adjustment to expected standards as a result of the self-assessment process that preceded AE-PMAQ-AB. This process was a component of both the PMAQ and the QualiAB programs.

PMAQ was the first institutional program of PHC facility assessment that covered the entire Brazilian territory, involving a large number of *in loco* AE-PMAQ-AB interviewers. In this process, transportation difficulties arose from the great distances between municipalities. Municipalities with large rural areas also proved difficult to reach. Thus, journeys frequently took hours or days, by different routes—air, river, or land. Roads were often precarious, and weather incidents, such as rainy seasons, blocked roads or isolated PHC facilities in the state of Amazonas [31–33]. Brazil’s great territorial extension and geographic diversity make *in loco* data collection surveys difficult and costly in many respects. Additionally, the high cost of the whole process of organization, selection, training, and hiring of a large number of professionals must be taken into account when choosing the best form of data collection [7].

Information and Communications Technology (ICT) has long been gaining ground in the health sector, with the incorporation of telemedicine technologies that make it possible to expand patient service and health care professionals’ training and support [34], as well as improving information record. As of 2020, with the measures to prevent the spread of Covid-19, the use of ICTs and computer equipment has been amplified in Brazil [35], which increases its potential for use in health care services and assessment processes.

Web-based structured assessments are limited by the number of questions that can be asked, which also limits the scope of the assessment and requires the selection of high-sensitivity and -specificity indicators. Additionally, this type of assessment requires great investment in establishing partnerships that will participate actively in and be committed to the assessment, thus yielding high response rates [7]. In-person assessments, on the other hand, even when based on structured questionnaires, make it possible not only to expand and diversify the subjects and interviewed professionals, but to observe the facilities directly and to use various instruments, such as semi-structured interviews with patients.

Some limitations of this study worthy of pointing out are the difference in subjects addressed by the two instruments, the time gap between the surveys, and their different levels of institutionalization and ability to induce participation. The AE-PMAQ-AB assessment was part of a financial incentive-granting evaluation program of the Brazilian Ministry of Health to improve service quality, whereas the QualiAB program was rather a self-assessment opportunity for the participating facilities.

On the other hand, web-based assessments, such as the one conducted through the QualiAB system, in addition to not posing the challenges of *in loco* data collection, can provide immediate access to results, reports and orientation to the system’s users. Quick access to results make them more likely to be used by the participants, particularly their direct users—the PHC teams–, thus allowing the assessment to complete all the stages of an assessment process.

Timely disclosure of results and good strategies for communicating them are mentioned in the literature [36, 37] as factors that promote knowledge and use of the assessments to underpin political decision making, redesign measures, and allocate financial resources [38, 39]. They also increase acceptance of the assessment, according to Rissi, Sager (2013) [40]. Another advantage is that this format does not interfere in the routine health care service, as it makes it possible to save the information record in case it is necessary to interrupt the response process. We can also add the ease of answering the questionnaire in a partial and scheduled way, which favors the involvement of a larger number of professionals in the discussion of the answers [7, 17].

The high level of concordance found between both assessments points to advantages in the use of web-based assessment instruments. These advantages become even more pronounced in light of the need for investments to expand assessment surveys, amplify the assessment culture, navigate pandemic scenarios, and further computerize PHC services, thus highlighting the importance of investing in web-based assessments as one more tool to improve the quality of PHC services and facilities.

## Supporting information

S1 Data (XLSX)
